# Orthopedic surgeons’ views on the osteoporosis care gap and potential solutions: survey results

**DOI:** 10.1186/s13018-019-1103-3

**Published:** 2019-03-06

**Authors:** David W. Barton, Daniel C. Griffin, Jonathan J. Carmouche

**Affiliations:** 1 Virginia Tech Carilion School of Medicine, 2 Riverside Circle, Roanoke, VA 24016 USA; 2 Department of Orthopaedic Surgery, Carilion Clinic, 2331 Franklin Rd SW, Roanoke, VA 24014 USA

**Keywords:** Fracture prevention, Fragility fracture, Osteoporosis, Quality improvement, Protocol, Fracture liaison service, FLS, Survey

## Abstract

**Introduction:**

Osteoporosis is often not recognized until one or more fractures occur, yet post-fracture screening remains uncommon. Orthopedic surgeons are well situated to address this care gap. Both a protocol-based approach and fracture liaison services (FLS) have been proposed. The present surveys assess orthopedists’ attitudes to these alternative models for addressing this care gap.

**Methods:**

Two digital surveys were sent to all orthopedic surgeons and orthopedic midlevel providers at a large level 1 trauma center 1.5 years apart.

**Results:**

Thirty-six of 47 survey recipients (77%) responded to the first survey; all 55 recipients (100%) responded to the second. Respondents recognized the importance of osteoporosis care, the inadequacy of current measures, and the potential of orthopedic surgeons to help address this gap. Respondents reported regular encounters with fragility fracture patients but limited familiarity with core aspects of osteoporosis screening and treatment, especially pharmacotherapy. While some respondents (40%) reported willingness to attempt a protocol-based approach to addressing this care gap, many others expressed reservations (60%) and support for a FLS-based approach was much higher (95%).

**Conclusions:**

A fracture liaison service model best fits the observed attitudes of orthopedic surgeons at this level 1 trauma center relative to a protocol-based approach. Protocol-based approaches may be preferable in alternate settings.

**Electronic supplementary material:**

The online version of this article (10.1186/s13018-019-1103-3) contains supplementary material, which is available to authorized users.

## Introduction

Osteoporosis is a major cause of disability worldwide through its association with fractures. Up to 50% of women and 20% of men will experience a fragility fracture at some point in their lives [1]. Fragility fractures, also known as minimal trauma fractures (MTF), are defined as fractures resulting from a fall from standing or an equivalent low-energy mechanism. Osteoporosis patients are frequently not recognized until they have experienced one or more fractures. Orthopedic surgeons are positioned to diagnose osteoporosis in fracture patients and to initiate appropriate screening, fracture care, and definitive osteoporosis treatment in those individuals.

US Medicare data indicate that 65% of women between 65 and 85 years of age who sustain a fracture are neither worked up nor treated for osteoporosis within 6 months of this fracture [2]. Two models of systematic interventions have been proposed to improve secondary prevention of osteoporotic fractures.

In the first model, a protocol is introduced to encourage orthopedic surgeons to assess patients for osteoporosis following fracture and initiate treatment as indicated [5, 6]. This model benefits from relatively low entry barriers as the orthopedist(s) may introduce the protocol with relative ease and without initially hiring additional personnel. Several articles have been published in the orthopedic literature suggesting strategies and protocols for orthopedic surgeons to use to improve their screening and initial treatment of osteoporosis in fracture patients [7–9].

The other model relies on the use of dedicated healthcare professional to evaluate patients for osteoporosis following fracture and initiate treatment as indicated. This model is commonly known as the fracture liaison service (FLS). The FLS model has shown excellent results at many institutions but has significant barriers to implementation [10–12].

The available data make clear that osteoporosis screening and treatment post-fracture represent a major gap in care within the orthopedic scope of practice and some sort of systematic intervention is necessary to manage it. The present surveys sought to determine the relative suitability of a protocol-based versus FLS approach to address this care gap at a large tertiary care center. No surveys have previously been published examining orthopedic surgeons’ attitudes towards these alternative models for osteoporosis management.

## Methods

Two electronic surveys were sent to all orthopedic surgeons and dedicated orthopedic midlevel providers at a busy level 1 trauma center in the USA. The first survey was sent via email in late October 2015, the second in April 2017. Responses to each survey were allowed over a 3-week period. Two weekly reminder emails were sent in each case.

The first survey consisted of seven questions, the second 21. Responses to each survey were assessed using descriptive statistics and figures. The text of each survey is included in Additional file 1. Survey results were collected and tabulated through a commercial survey service [13].

## Results

Thirty-six of the 47 survey recipients (77%) responded to the first survey (survey #1). All 55 responded (100%) to the second survey (survey #2). Table 1 reports recipient and respondent characteristics for both surveys.

**Table 1:** Recipient and respondent characteristics for both surveys

	Survey #1 (respondent/recipient)	Survey #2 (respondent/recipient)
Respondents	36/47	55/55
Physician	28/34	38/38
MD/DO orthopedic surgeon	24/28	35/35
Orthopedic traumatologist	4/4	4/4
Podiatrist (DPM)	4/4	3/3
Non-operative orthopedist	1/2	2/2
Midlevel providers (orthopedic)	8/13	17/17
PA (orthopedic)	8/12	16/16
NP (non-operative orthopedic)	0/1	1/1

**Fig. 1:**
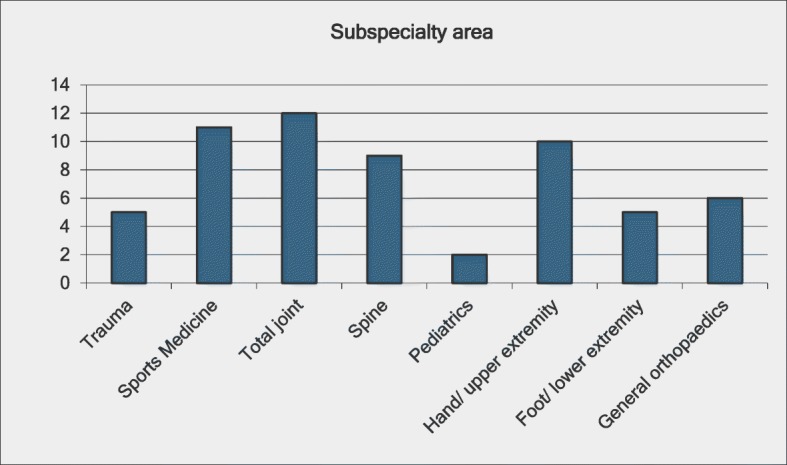
Respondents’ areas of specialization based on responses to the second survey. Information on subspecialty area was not collected in the first survey. Three respondents selected multiple areas of specialization

Survey #2 includes several questions on respondent demographics including area of specialization, years in practice, and frequency of call. Respondents’ areas of specialization are depicted in Fig. 1. Forty percent of respondents had been in practice for 5 years or less, 31% for 6 to 15 years, and 29% for more than 15 years. Thirty-five percent of respondents did not take call. Fifty-one percent of respondents took call once per month or more, and 14% took call less than once per month. Survey #2 included two questions about respondents’ exposure to minimal trauma fracture patients. Most respondents (33, 60%) believed they saw five or fewer minimal trauma fracture (MTF) patients per week, 13 respondents (24%) thought they saw 6–10 MTF patients, 8 thought they saw 11–20 (15%), and 1 respondent saw more than 20. Most respondents (43, 78%) believed they spent less than 1 h per week on MTF patients, while 11 respondents (20%) spent 1–3 h, and 1 respondent spent more than 3 h.

**Fig. 2:**
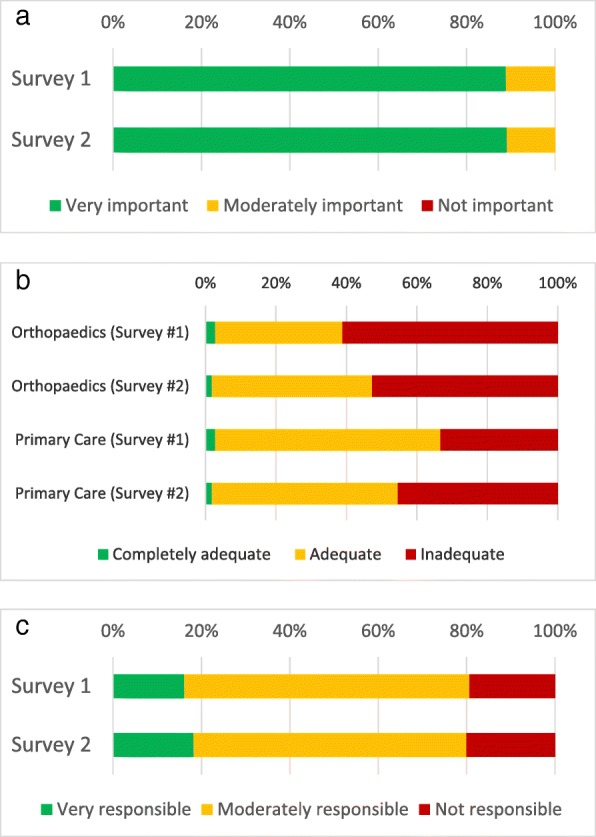
**a** Respondents’ perceptions of the importance of osteoporosis care. **b** The perceived adequacy of osteoporosis care by setting. **c** How responsible respondents felt for initiating osteoporosis care

Both surveys assessed respondents’ views on the importance of osteoporosis care, adequacy of currently provided care, and sense of ownership over osteoporosis management, as displayed in Fig. 2.

**Fig. 3:**
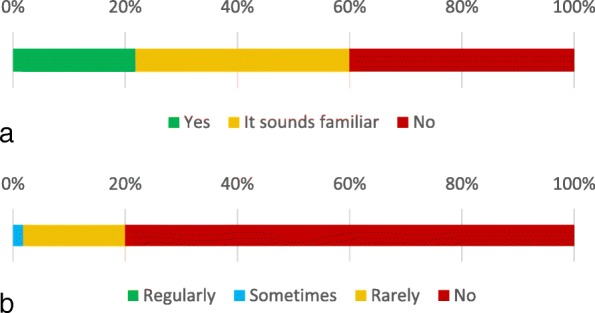
**a** Depiction of whether respondents knew what the FRAX score is. **b** Depiction of the frequency of FRAX score use in respondents’ clinical practice

Survey #2 evaluated respondents’ familiarity with various aspects of osteoporosis management. Respondents’ familiarity with and use of the Fracture Risk Assessment Tool (FRAX) score is displayed in Fig. 3.

**Fig. 4:**
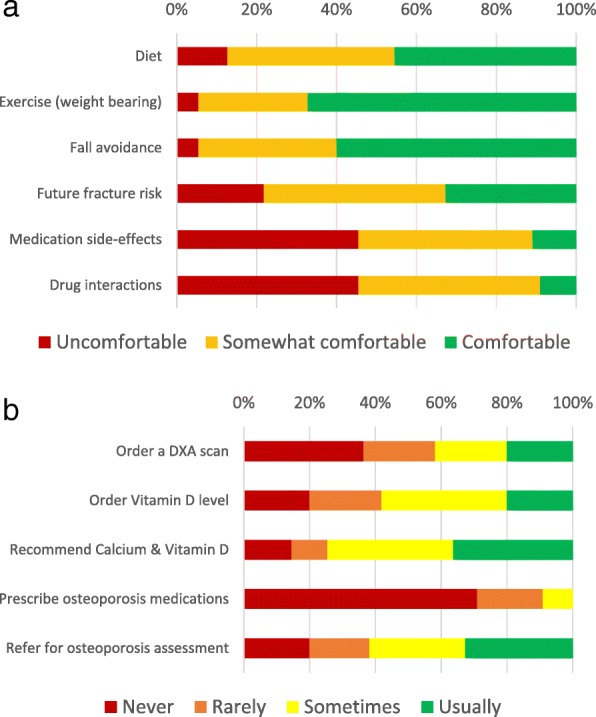
**a** Respondents’ self-reported comfort with providing patient guidance on various aspects of osteoporosis care. **b** Respondents’ self-reported frequency of osteoporosis management steps in MTF patients

**Fig. 5:**
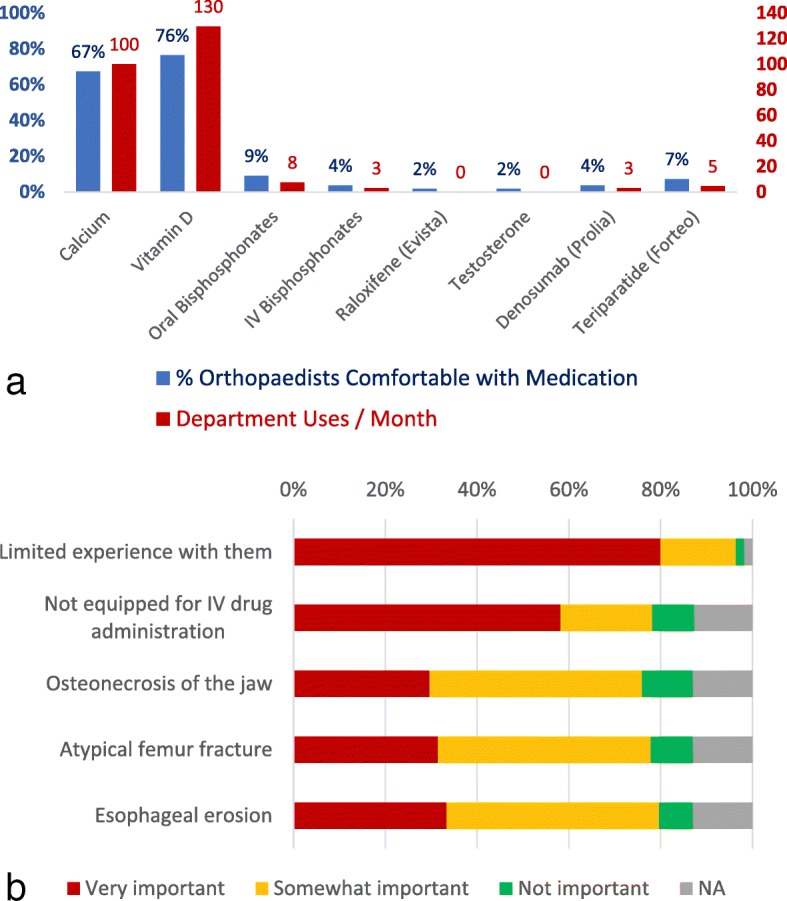
**a** A summary of prescriber comfort with and self-reported use of different osteoporosis medications when summed across all providers in the department. **b** Respondents’ self-reported reasons why survey respondents felt uncomfortable prescribing medications

Figure 4a shows providers’ self-reported comfort with providing patient guidance on various aspects of osteoporosis care. Figure 4b shows self-reported frequency of osteoporosis management steps in MTF patients. Figure 5a shows a summary of prescriber comfort with and self-reported use of different osteoporosis medications when summed across all providers in the department. Figure 5b shows self-reported reasons why survey respondents felt uncomfortable prescribing medications. It revealed the greatest indication for not prescribing a therapy was limited experience with that therapy.

**Fig. 6:**
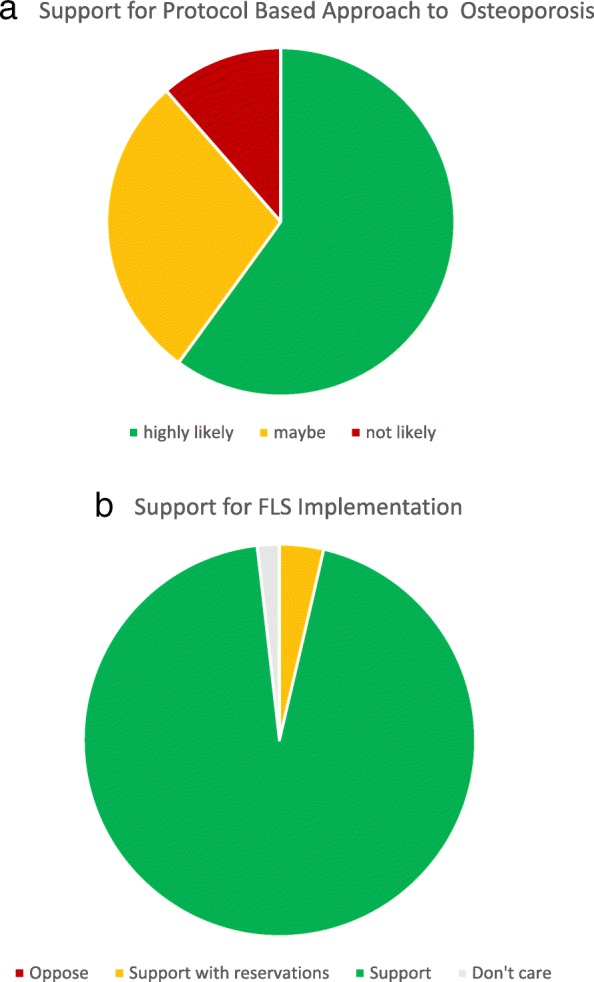
**a**, **b** Respondents’ attitudes towards either a protocol-based or FLS-based approach to osteoporosis management

Recipients of survey #1 were asked, “If an established protocol was given to you, how likely are you to follow the protocol if it includes ordering DXA scans and writing the initial prescriptions for osteoporosis medications?” Responses are displayed in Fig. 6a. Forty percent of respondents either refused (11%) or expressed some reluctance (29%) to follow a protocol intended to help orthopedic surgeons better recognize and initiate management of osteoporosis. In contrast, 53 respondents (95%) to survey #2 supported initiation of a fracture liaison service to evaluate and manage low bone mass in MTF patients. Two others (4%) support the initiative with reservations, and only one person was indifferent. No one opposed the establishment of the FLS (Fig. 6b). Respondents were significantly more likely to support an FLS versus a protocol-based approach (OR 11.56, 95% CI 3.01–44.40).

**Fig. 7:**
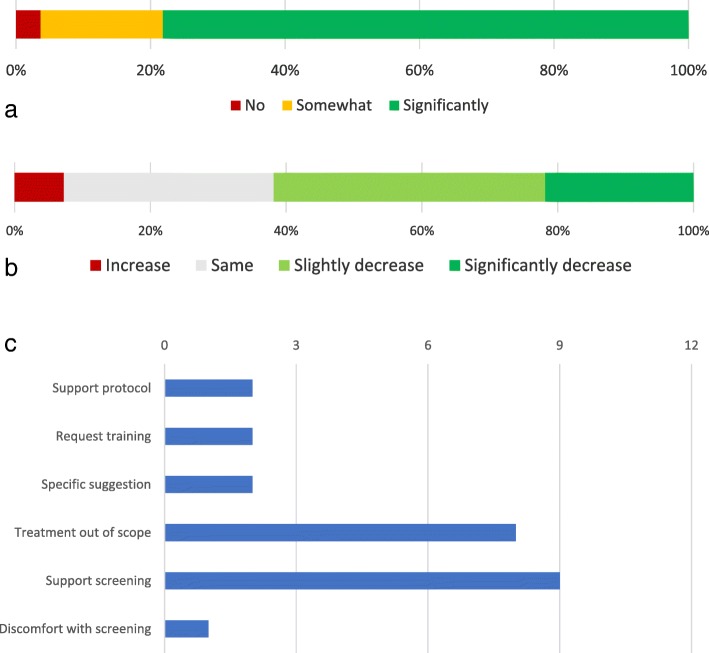
**a** Depiction of respondents’ perceptions of whether having access to a dedicated bone health ACP (NP, PA) would improve patient care. **b** Depiction of the anticipated impact of adding a bone health ACP on respondents’ workloads. **c** Free-text response themes to survey #1 (*n* = 13)

Figure 7a and b depict respondents’ perceptions about the impact of having a dedicated advanced care provider (ACP), such as an NP or PA, facilitating osteoporosis care in the orthopedic setting following MTF on patient care and respondents’ workloads. Figure 7c shows free-text response themes to survey #1. Free-text responses to survey #2 were less common (7/55 vs 13/36). They tended to focus on general statements of support and specific suggestions for improvements to the FLS plan.

## Discussion

Orthopedic surgeons at a large level 1 trauma center with a high volume of osteoporotic fracture care overwhelmingly recognized the importance of osteoporosis. This is reassuring as it suggests the surveyed orthopedic surgeons take osteoporosis seriously as a disease entity. They recognized that post-fracture osteoporosis care is imperfect in both the orthopedic and primary care setting, suggesting that they recognize the post-fracture osteoporosis care gap. Because orthopedic surgeons recognized the magnitude of the post-fracture osteoporosis care gap, we were able to assess orthopedic surgeons’ willingness to commit to either a protocol-based or FLS-based approach without concern that lack of comprehension might hinder their commitment to either solution.

The surveyed orthopedic surgeons endorsed feeling moderately responsible for osteoporosis care. That finding fits with our later observations that in the free-text responses, orthopedic surgeons generally endorsed taking an increased role in osteoporosis screening, but most were uncomfortable assuming a more active role in pharmacotherapy for osteoporosis. On the ranked choice scale, many orthopedists expressed unfamiliarity with how to approach osteoporosis management and most said they were uncomfortable prescribing medications for osteoporosis, further reinforcing the observation that many orthopedists are uncomfortable assuming direct responsibility for initiating osteoporosis care. This is echoed by the finding that only 22% of respondents knew what the FRAX score is, although 38% thought it sounded familiar, and 80% never use it in clinical practice. Although the FRAX score is used more for primary screening than evaluation after fracture, where orthopedic surgeons are more commonly involved, respondents’ lack of familiarity with this common component of osteoporosis evaluation demonstrates the knowledge gap they profess. Further, when asked about their concerns regarding starting osteoporosis medications, they commonly cited atypical femur fractures and osteonecrosis of the jaw. These side-effects, while real, are heavily outweighed through fracture prevention. This finding is consistent with a larger pattern of hyper-awareness of these side-effects with regard to bisphosphonate use in the osteoporosis population. While these side-effects do occasionally occur with oral bisphosphonates and may also occur with denosumab, they are rare at osteoporosis doses, occurring more often when used as part of chemotherapy. This misperception is thought to have contributed to a decline in the treatment rate of osteoporosis [14]. The potential for pill esophagitis with oral bisphosphonates was also commonly cited and is a legitimate concern in the local population, as gastroesophageal reflux disease is highly prevalent locally and increases the risk of this relatively prevalent side-effect. The most commonly cited reason was lack of familiarity, which is a good reason in the short term given the potential for drug-drug interactions and adverse effects. While it is possible to address some of these knowledge gaps through educational interventions, such interventions should be targeted to most efficiently improve clinical care. Given the knowledge gaps documented here, it is likely more efficient to target such educational interventions to focus on identifying which patients need further work up, initial orders for work up, and where to refer them. Twenty-seven percent of respondents said one of their leading reasons for not providing better osteoporosis care was because doing so was not their responsibility. Their rejection of the role in which a post-fracture osteoporosis protocol would place them and general discomfort with the critical task of initiating pharmacotherapy for osteoporosis within such a protocol suggested that implementing a protocol to address the post-fracture osteoporosis care gap might not represent the best solution at this level 1 trauma center. When asked directly whether they supported a protocol-based approach to addressing the post-fracture osteoporosis care gap, 40% of respondents were either unsure of whether they would follow it or openly rejected it.

Based in part on these results, the Department of Orthopaedic Surgery opted to pursue the objective of improving osteoporosis recognition and management post-fracture by using a fracture liaison service (FLS) model instead of a protocol-based approach. As expected, this approach was significantly more time consuming to implement at 20 months from initial plan to first day of clinic versus an expected 6 months for a protocol-based approach with paired lecture series. The FLS conforms better to the physician preferences observed in our survey since it calls on orthopedic surgeons to refer patients with suspected fragility fractures to our fracture liaison service, where a nurse practitioner formally evaluates them for osteoporosis and initiates treatment as indicated. Because this concentrates the workup and management in the hands of a non-surgeon, it alleviates the burden of initiating medical management for osteoporosis that the orthopedic surgeons would have had under the protocol. The FLS model also benefits from economies of scale in that the nurse practitioner running it is dedicated to bone health assessments and management in the post-fracture period and thus quickly becomes experienced in this domain. Other FLS models rely on a nurse coordinator to orchestrate dual-energy X-ray absorptiometry (DXA) orders and referral to an outside provider for evaluation and management. We selected a model with an in-house nurse practitioner over a purely coordinator-based approach in part because of the scarcity of endocrinologists, rheumatologists, and primary care providers with an interest in osteoporosis in our area. Given all these factors, most of which we believe generalize, we encourage implementation of FLS programs wherever feasible.

Our conclusion is consistent with the recommendations of professional bodies including the International Osteoporosis Foundation (IOF), American Orthopaedic Association (AOA), and American Society of Bone Mineral Research (ASBMR), which all strongly endorse the FLS model as the preferred approach to secondary prevention of osteoporotic fractures [3, 4, 15].

However, the FLS model is not feasible in all settings. The logistical and institutional political barriers to implementing an FLS can be extensive. A major impediment is the need to hire dedicated personnel. New organizational structures and billing models may also need to be completed prior to implementation. These steps take time which may delay introduction of an intervention. Resource-limited settings may have difficulty marshaling the initial resources to establish an FLS, despite their tendency to be financially self-sustaining in the long term. Smaller private practices and independent surgeons are unlikely to have the resources to establish even a minimal, nurse coordinator-based FLS.

These factors may help explain why FLS centers are still relatively sparse, despite widespread calls to implementation by professional bodies and in the literature. The distribution of FLS centers can be visualized using the map of IOF Capture the Fracture certified centers [4]. The large gaps in that map demonstrate the scarcity of official FLS programs throughout much of the world, even in relatively affluent nations such as the USA and Canada. Given these barriers, some institutions may opt to pursue a protocol-based approach to address this care gap.

## Conclusions

This study demonstrates that orthopedic surgeons at this institution recognize the importance of osteoporosis care and the existence of a care gap whereby patients presenting with fragility fractures are often not evaluated or treated for osteoporosis. They wish to provide better care. They support an increasing role for orthopedic surgeons in screening for osteoporosis, but many expressed reservations about taking responsibility for initiating osteoporosis treatment and endorsed related knowledge gaps. These responses helped guide us to establish a fracture liaison service rather than pursue a protocol-based approach to addressing the osteoporosis care gap at our institution. For orthopedists without ready access to the resources to hire personnel to establish a fracture liaison service, protocol-based approaches remain useful interventions.

## Additional file

Additional file 1: Survey text. (DOCX 20 kb)

## Abbreviations

FLS: Fracture liaison service
MTF: Minimal trauma fracture
DXA: Dual-energy X-ray absorptiometry
MD: Medical doctor
DO: Doctor of osteopathy
DPM: Doctor of podiatric medicine
