# Supplementary material for: Orthopedic surgeons’ views on the osteoporosis care gap and potential solutions: survey results
Source: J Orthop Surg Res. 2019 Mar 6;14:72. doi: 10.1186/s13018-019-1103-3 (PMC6402163; doi:10.1186/s13018-019-1103-3)
Supplement: Supplementary file 1 — Survey text. (DOCX 20 kb) [file 13018_2019_1103_MOESM1_ESM.docx]

**Additional file 1**

**Survey #1**

Credentials

- MD/DO
- DPM
- PA
- NP

Do you believe that Osteoporosis care is important?

- Very important
- Moderately important
- Not important

Do you believe you, as the Orthopaedic Surgeon, is [*SIC*] responsible for initiating osteoporosis treatment?

- Very responsible
- Moderately responsible
- Not responsible

Do you believe that the primary care team (PCPs, FNPs, Primary care PAs, etc) provides adequate care for osteoporosis patients?

- Completely adequate
- Adequate
- Inadequate

Do you believe you, as the Orthopaedist, provide adequate care for osteoporosis patients?

- Completely adequate
- Adequate
- Inadequate

Rank the reasons why you do not provide completely adequate osteoporosis care.

- Not my responsibility
- Knowledge gap, i.e. not sure what exactly to do
- Uncomfortable with medications
- No time
- I provide completely adequate osteoporosis care

If an established protocol was given to you, how likely are you to follow the protocol if it includes ordering DEXA scans and writing the initial prescriptions for osteoporosis medications?”

- Not likely
- Maybe
- Highly likely

Comments:

**Survey #2**

Credentials

- MD/DO
- DPM
- PA
- NP
- Other

Subspecialty area

- Trauma
- Sports Medicine
- Total joint
- Spine
- Pediatrics
- Hand/ upper extremity
- Foot/ lower extremity
- General orthopaedics
- Other (please specify)

Do you take call?

- No
- Less than once per month
- More than once per month

Years in practice since completing training (e.g. after fellowship)?

- 0-5
- 6-15
- 16+

Do you believe that Osteoporosis care is important?

- Very important
- Moderately important
- Not important

Do you believe you, as the Orthopaedic Surgeon, are responsible for initiating osteoporosis treatment?

- Very responsible
- Moderately responsible
- Not responsible

Do you believe that the primary care team (PCPs, FNPs, Primary care PAs, etc) provides adequate care for osteoporosis patients?

- Completely adequate
- Adequate
- Inadequate

Do you believe you, as the Orthopaedist, provide adequate care for osteoporosis patients?

- Completely adequate
- Adequate
- Inadequate

How many patients per week do you see for management of low energy fractures?

- 0-5
- 6-10
- 11-20
- 21-30
- >30

How many hours per week do you spend evaluating or managing their bone mass (osteoporosis, osteopenia)?

- 0-1
- 1-3
- 3-5
- 5+

Do you know what the FRAX score is?

- Yes
- It sounds familiar
- No

Do you use the FRAX (Fracture Risk Assessment Tool) score in your routine clinical practice?

- No
- Rarely
- Sometimes
- Regularly

In low energy fracture patients without prior treatment, how often do you do each of the following? (Never, Rarely, Sometimes, Usually)

- Order a DXA (bone mineral density) scan, if not done in previous 2 years
- Order a Vitamin D level
- Recommend or prescribe Calcium and Vitamin D supplementation
- Prescribe medications for low bone mass
- Refer them for low bone mass assessment and management

How many times per month do you prescribe each of the following medications for low bone mass?

(0, 1-2, 3-5, 6+)

- Calcium
- Vitamin D
- Oral Bisphosphonates
- IV Bisphosphonates
- Raloxifene (Evista)
- Testosterone
- Denosumab (Prolia)
- Teriparatide (Forteo)
- Other (please specify)

How comfortable do you feel prescribing each of the following medications for low bone mass?

(Uncomfortable, Somewhat uncomfortable, fairly comfortable, comfortable)

- Calcium
- Vitamin D
- Oral Bisphosphonates
- IV Bisphosphonates
- Raloxifene (Evista)
- Testosterone
- Denosumab (Prolia)
- Teriparatide (Forteo)

For those with which you are uncomfortable prescribing, what makes you uncomfortable?

(N/A, Not important, Somewhat important, Very important)

- Limited experience with them
- Not equipped for IV drug administration
- Osteonecrosis of the jaw
- Atypical femur fracture
- Esophageal erosion

How comfortable do you feel providing specific guidance to patients with low bone mass regarding the following topics?

(Uncomfortable, somewhat comfortable, comfortable)

- Diet
- Exercise (weight bearing)
- Fall avoidance
- Drug interactions
- Medication side-effects
- Future fracture risk

Would having access to a dedicated advanced care provider (NP, PA) responsible for evaluating and managing osteoporosis in patients presenting with minimal trauma fracture [*SIC*] improve your management of such patients?

- No
- Somewhat
- Significantly

Comments:

How do you expect having such a provider would impact the time you spend managing bone mass?

- More work
- Same
- Slightly decrease
- Significantly decrease

Comments:

Do you support the department’s effort to add a dedicated advanced care provider with responsibility for evaluating and managing low bone mass in low energy fracture patients (the FLS service)?

- Oppose
- Support with reservations
- Support
- Don’t care

Comments:

Do you have any other thoughts or concerns about osteoporosis care?
